# InteMAP: Integrated metagenomic assembly pipeline for NGS short reads

**DOI:** 10.1186/s12859-015-0686-x

**Published:** 2015-08-07

**Authors:** Binbin Lai, Fumeng Wang, Xiaoqi Wang, Liping Duan, Huaiqiu Zhu

**Affiliations:** 1 State Key Lab for Turbulence and Complex Systems and Department of Biomedical Engineering, College of Engineering, Peking University, Beijing, 100871 China; 2 Center for Quantitative Biology, Peking University, Beijing, 100871 China; 3 Department of Gastroenterology, Peking University Third Hospital, Beijing, 100191 China

## Abstract

**Background:**

Next-generation sequencing (NGS) has greatly facilitated metagenomic analysis but also raised new challenges for metagenomic DNA sequence assembly, owing to its high-throughput nature and extremely short reads generated by sequencers such as Illumina. To date, how to generate a high-quality draft assembly for metagenomic sequencing projects has not been fully addressed.

**Results:**

We conducted a comprehensive assessment on state-of-the-art de novo assemblers and revealed that the performance of each assembler depends critically on the sequencing depth. To address this problem, we developed a pipeline named InteMAP to integrate three assemblers, ABySS, IDBA-UD and CABOG, which were found to complement each other in assembling metagenomic sequences. Making a decision of which assembling approaches to use according to the sequencing coverage estimation algorithm for each short read, the pipeline presents an automatic platform suitable to assemble real metagenomic NGS data with uneven coverage distribution of sequencing depth. By comparing the performance of InteMAP with current assemblers on both synthetic and real NGS metagenomic data, we demonstrated that InteMAP achieves better performance with a longer total contig length and higher contiguity, and contains more genes than others.

**Conclusions:**

We developed a *de novo* pipeline, named InteMAP, that integrates existing tools for metagenomics assembly. The pipeline outperforms previous assembly methods on metagenomic assembly by providing a longer total contig length, a higher contiguity and covering more genes. InteMAP, therefore, could potentially be a useful tool for the research community of metagenomics.

**Electronic supplementary material:**

The online version of this article (doi:10.1186/s12859-015-0686-x) contains supplementary material, which is available to authorized users.

## Background

Without the need for prior laboratory cultivation, metagenomics, as the study of sequence data directly from microbial communities in their natural habitats, has shown great power in investigating ubiquitous microorganisms that have intimate relationships with human beings as well as all other living organisms [1–3]. Recently, the next-generation sequencing (NGS) technology has greatly facilitated metagenomics, such as the launch of the Human Microbiome Project [4] and the MetaHIT project [2], providing numerous metagenomic data that can be mined for information on the compositional and functional properties of human microbial communities. Moreover, the drastic reduction in the cost of sequencing has allowed more and more laboratories to initiate metagenomic sequencing projects to decode complex microbiomes.

In order to carry out the analysis of the metagenomes, such as gene-finding and binning [5–8], short reads (*e.g.* 100 bp for Illumina reads) are usually expected to be assembled first into longer contiguous sequences called contigs (with sizes ranging from several hundreds of bps to the whole chromosome) to provide more valuable genomic content. Despite of early endeavours to assemble reads from Sanger and 454 sequencers, such as Genovo [9], Xgeovo [10] and MAP [11], high-throughput NGS short reads raise new challenges for this problem. The challenges come from the situation of high-throughput and extremely short reads generated by such as Illumina sequencers, as well as from intrinsic complications of metagenomic data caused by the microbial communities. Except for a few studies, most metagenomes using NGS remain unassembled and have been deposited directly into public databases. As part of the MetaHIT project, Qin et al. [2] assembled metagenomic short reads from 127 human microbial samples and reported a catalogue of microbial genes from the intestinal tract. However, the assembler they employed, SOAPdenovo [12], is not designed specifically for metagenome assembly but only for isolated genomes. Another single-genome assembler, Velvet [13], has also been used to assemble permafrost metagenomes [14]. Recently, several assemblers for metagenomic short reads, such as IDBA-UD [15], MetaVelvet [16], Ray [17], and SPAdes [18, 19], are reported to be superior to other methods. In addition, CABOG, previously named Celera Assembler and employed in both single genome and metagenome assembly tasks, has been rebuilt to support Illumina reads in its latest version [20, 21]. Recently, a metagenomic assembler, Omega, has been reported to outperform others mainly on long reads data (MiSeq 300 bp datasets) [22].

Despite the aforementioned progress, metagenomic sequence assembly is still regarded as a challenge, both with respect to the data and the assembly process. It is known that metagenomic data contain sequences from so many species within a community, with many differences in abundances, levels of polymorphisms, and relationships with each other [23]; In addition, other technical factors also affect the assembly quality, such as the depth of sequencing coverage, the sequencing errors, or the choice of *k*-mer size for assembling algorithm [11, 24]. Consequently, using these tools may be placed many more limitations than we might think. That is, they may not suit all the species in the metagenome data, or not be able to deal with all of the assembly issues. To this end, it is useful for the scientific community to realize the properties of existing assemblers with their performance on real metagenomic NGS data. Furthermore, it is also important if we can improve assembly quality by combining the strengths of these tools to deal with the complex task of metagenomic assembly. It has been reported that assembly reconciliation which merges different draft genome assemblies improved the assembly quality [25–27], especially in the task of metagenomic assembly [28]. However, these studies on assembly reconciliation only provided general merging algorithm, leaving the problem of deciding which assemblers to choose unresolved.

In this work, we first conducted a comprehensive assessment on state-of-the-art *de novo* assemblers including both *de Bruijn* graph based and overlap graph-based assemblers, with simulated metagenomic NGS data. In contrast to previous metagenomic assembly evaluation efforts that deemed the community as a whole [29, 30], we evaluated the assemblers by investigating how they perform on individual species in terms of factors such as sequencing depth of coverage and/or genomic similarity within the microbial community. Such a strategy has enabled us to reveal that different computational strategies not only complement each other by having different advantages in assembling sequences with different levels of sequencing depth, but also complement each other on assembling the sequences from the same genomes. Based on these findings, we further developed a *de novo* pipeline, named InteMAP, which integrates three tools, ABySS [31], IDBA-UD [15], and CABOG [21], for metagenomic assembly. Taking advantage of the strength of each assembler and the complementary nature among them, the InteMAP pipeline shows remarkable improvement in metagenomic assembly. Unlike other metagenomic assembly pipeline, such as MetAMOS [32] and MOCAT [33], which directly provide a collection of available assemblers to users without a smart solution according to input short reads data, the InteMAP pipeline generates improved assembly based on integrating selected assemblers. The tests upon both simulated and real metagenomic NGS data show InteMAP has the potential for the assembly of metagenomics.

## Results

### Evaluation of assemblers revealing their respective advantages

In order to understand how state-of-the-art *de novo* assemblers perform on metagenomic datasets with short DNA reads, here we conducted the evaluation of several leading assemblers currently in wide use. Five latest assemblers, ABySS [31], CABOG [21], IDBA-UD [15], MetaVelvet [16], and SOAPdenovo [12], were selected in the current study. Among them, ABySS, CABOG, and SOAPdenovo are general-purpose genome assemblers while the other two are specifically designed for assembling metagenomic reads. For the benchmark data, we constructed a simulated dataset consisting of 113 microbial species with different abundances, called *sim-113sp*, to model a real complex metagenome. For each assembler, we ran the program by multiple options and selected the best results (see Methods and Additional file 1: Supplemental Methods for details).

It should be pointed out that our evaluation of these assemblers aims to examine their performance directly based on the details of reads, including the sequencing depth of coverage. Fortunately via simulated metagenome data, we may trace the details by mapping each read or contig into the corresponding source genome. Thereby, each assembly is applied to each individual genome within the simulated community. Herein, we evaluated three common-use measurements as contiguity, completeness, and correctness of each assembly for individual genome by calculating correct *N*50 size, assembly cover rate, and structural assembly errors in contigs (see Methods for how to calculate correct *N*50). Furthermore, assembly cover rate is defined as the proportion of the reference genome covered by contigs. Structural assembly errors are defined as in previous studies, including “mis-joins” of two unrelated sequences and segmental indels in the contigs [34].

We have noticed that each assembler provides similar performance for genomes of similar sequencing coverage. Therefore, 113 species within the *sim-113sp* dataset are classified into three groups by their sequencing depth levels: high (≥30× depth), medium (15-30×) and low coverage (≤15×). From the results of CABOG and IDBA-UD, we selected those that have the best trade-off between contiguity and correctness on most species. For the assemblers using the *k*-mer graph-based approach (ABySS, MetaVelvet, and SOAPdenovo), we found that assemblies configured with lower *k*-mer sizes have the better performance at lower coverage levels, while those with higher *k*-mer sizes present better performance at higher coverage levels. Thus, at different levels of sequencing coverage, we chose different *k*-mer sizes for the assemblies. Specifically, we selected the assembly with *k*-mer size 23 for lower coverage levels (low and medium clusters) while 51 for higher levels (medium and high clusters). It should be noted here that selecting a close *k*-mer size does not significantly change the assembly outcome.

**Fig. 1:**
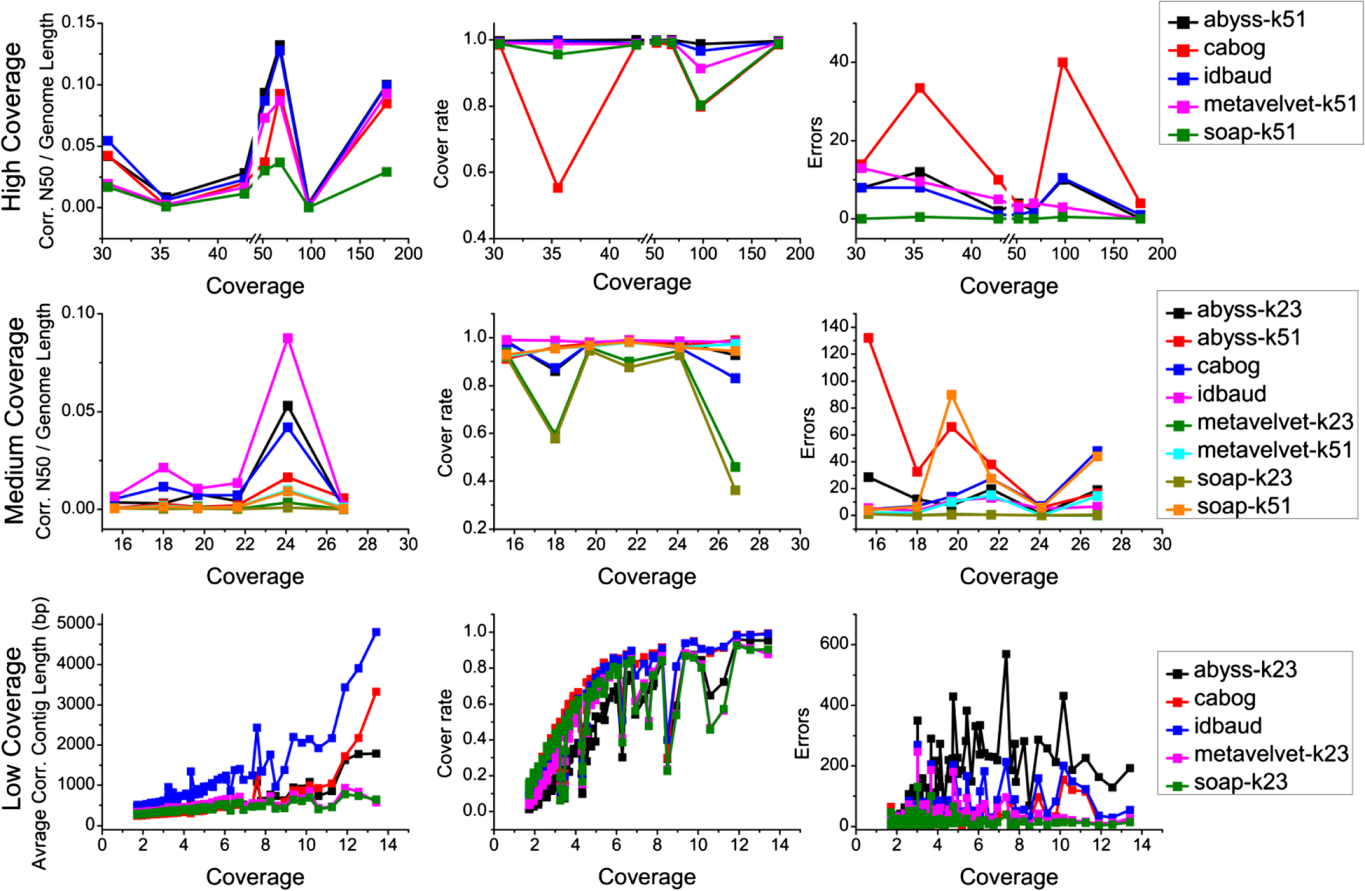
Assembly performances on the simulated metagenome dataset for the five assemblers. Assembly performances on the simulated metagenome dataset for the five assemblers (ABySS (*k*-mer size 51), CABOG, IDBA-UD, MetaVelvet (*k*-mer size 51), and SOAPdenovo (*k*-mer size 51)) are shown. The left column draws the ratio of correct *N*50 size to genome length, the medium column draws the assembly cover rate and the right column draws the assembly error counts of the assemblies. The top panel reports the performances for data at the high-coverage level (≥30×), while the medium panel does at the medium-coverage level (15-30×), and the bottom panel does at the low-coverage level (<15×)

As shown in Figure 1, at both high and medium coverage levels, assembly cover rate of most assemblies reaches or closely approaches saturation, so the correct *N*50 size of contigs is selected to evaluate the capability of each assembler. At low coverage level, we used assembly cover rate and average contig size for evaluation, because most assemblies have an extremely low correct *N*50 size, which even drop to 0 since the assembly cover rate is less than 50 %. We then sum the performance of each assembler at different sequencing coverage levels on both capability and accuracy from the results shown in Fig. 1 and Additional file 1: Figure S1. The results show that ABySS has the best performance with a trade-off between correct *N*50 size and errors at high coverage level, but the performance degenerates from ~20× as the coverage decreases with more errors and shorter contigs. IDBA-UD has the best performance at medium coverage level with the best trade-off between contig size and error rate, and a good performance (though not as good as ABySS) at high coverage level. At low coverage level, IDBA-UD achieves both high assembly cover rate and average length, but also generates many more errors. CABOG performs well at medium and low coverage levels but does poorly at high coverage level. Both MetaVelvet and SOAPdenovo generate fewer errors than the other assemblers, but also have shorter contigs and a lower assembly cover rate at all three coverage levels. These results demonstrate the respective advantages as well as disadvantages of the assemblers, which lead to them being suitable for different data depth of sequencing coverage. For example, ABySS has the best performance on high depth, while IDBA-UD has the best performance on medium and low depth.

**Fig. 2:**
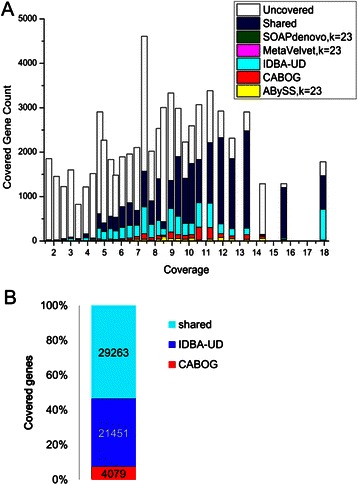
Genes covered by different assemblies. **a** The number of genes uncovered by any assemblies, covered by more than one assemblies and covered exclusively by only one assembly of the five assemblers (ABySS (*k*-mer size 23), CABOG, IDBA-UD, MetaVelvet (*k*-mer size 23), SOAPdenovo (*k*-mer size 23)), for the species with low coverage (<18×) are stacked. The lateral axis shows the coverage of each species. Only partial species with coverage lower than 18× are drawn. **b** The stacked bar plot draws the distribution of the total genes on species with low coverage (<18×) covered by IDBA-UD assembly and CABOG assembly. The *blue* part and the *cyan* part represent genes covered exclusively by CABOG and IDBA-UD, and the *magenta* part represents the genes shared by CABOG and IDBA-UD

We further analyzed the assemblies for the same species and investigated to what extent different approaches may complement each other. To address this, for each assembly on the *sim-113sp* dataset, we counted the genes that were completely contained in contigs. At lower coverage level (<20×), except for a large part shared by at least two assemblies, the genes covered by IDBA-UD exclusively occupies the largest proportion of all the covered genes, followed by CABOG (see Fig. 2a). However, as shown in Fig. 2b, IDBA-UD and CABOG share 53.4 % of the genes covered by them, while the percentage of the genes covered exclusively by IDBA-UD is 39.2 % and by CABOG is 7.4 %. This means that IDBA-UD and CABOG both miss a considerable number of the genes covered by the other assembly. In contrast, assemblies from ABySS, MetaVelvet, and SOAPdenovo have just a few genes missed by IDBA-UD assembly (see Additional file 1: Figure S2). The most likely reason is that ABySS, MetaVelvet, and SOAPdenovo use the same approach as IDBA-UD (*k*-mer graph), while CABOG uses a different one (overlap graph). At a higher coverage level (≥20×), almost all the genes are covered by at least two assemblers, except for a small proportion of genes in a few species that are exclusively covered by ABySS and IDBA-UD assembly (Additional file 1: Figure S3). This result indicates that most assemblies are able to cover the vast majority of genes at high coverage level.

We finally investigated whether the mixture of species impacts the metagenomic assembly for each assembler (details shown in Additional file 1: Supplemental Methods). As shown in Additional file 1: Figure S4 and Additional file 1: Figure S5, the test on four small synthetic datasets with mixed genomes demonstrates that the mixture of uneven coverage sequences did not significantly change the result of each individual genome in metagenomic assembly process, for most assemblers except ABySS (ABySS is found to greatly increase the errors). In contrast, the mixture of the closely related species had a significant impact on both correct *N*50 size and error count for most assemblers, with shorter *N*50 and more errors. Nonetheless, the impacts from the mixture of different species seem to be insensitive or quite similar for all the assemblers in this test. Therefore, the variation at specific coverage level in the metagenomic assembly of different assemblers are, to a large extent, determined by the assembly capabilities of assemblers at that level, rather than the abilities to resolve the impacts of the mixture of reads.

To sum up, our analysis revealed that any given individual assembler has bare means of providing the equal performance of assembly on short reads with different sequencing depth. Therefore, a user may rather be caught in trouble when using these tools for reads sequenced with heterogeneous depth as in a real metagenomic sample. However, on the basis of thoroughly understanding the assembling approaches, we expected that a fine-tune combinatorial application of these tools instead of running single tool would improve the assembly outcome.

### The InteMAP pipeline and its performance on simulated metagenomic dataset

As mentioned above, upon test of the *sim-113sp* dataset, we found that existing *de novo* assemblers achieve their best performances on reads with different sequencing depth levels, moreover they are expected as being complementary to each other. Based on these findings, we devised a pipeline for metagenomic *de novo* assembly, named InteMAP, to integrate three *de novo* assemblers, IDBA-UD [15], ABySS [31] and CABOG [21], which are shown to have their best performances at different depth levels and to be actually complementary to each other in our test. The InteMAP pipeline aims to generate a high-quality assembly on both high and low depth of coverage. In general, the pipeline begins with an algorithm to estimate the coverage of each read, and then integrates assemblies via two strategies: for different sequencing coverage reads, different optimal assemblers are used; while for reads of the same coverage, two optimal assemblers are used and then integrated. Iteratively, the pipeline integrates two assemblies at each step. For low coverage reads, the assemblies by IDBA-UD and CABOG are integrated. If high coverage reads are detected in the data, InteMAP combines the assemblies by ABySS and IDBA-UD for these sequences. Finally, the assemblies integrated from low and high coverage are further merged into the final assembly. Herein, the integration algorithm is specially designed for contigs produced by different assemblers. For non-overlapped contigs from two assemblies, the pipeline directly includes all the non-overlapped contigs, while for overlapped contigs from two assemblies, the pipeline merges the overlapped segments and makes a decision on the contradictory point, whether to extend from one contig or to cut at the point (see section Methods for detailed description of the algorithm).

We then report the merits of the integrated assembling pipeline upon tests described below. Unlike the evaluation of the five assemblers mentioned above, herein we are confronted with another situation. To apply an assembler, including our InteMAP pipeline, to practical assembling metagenomic data, a read cannot be indicated its source genome as well as sequencing coverage. Therefore, as in previous studies [11, 16, 35], the statistics of the assembly on the whole community other than the assemblies calculated by individual genomes are used to evaluate the performance of the assembler on simulated metagenomic data. The first measure of assembly we assessed is the total length of the reference sequences covered by contigs, named “total cover length”, from which we evaluated the capability of the assembler to generate contigs. We also evaluated contiguity of the assembly using the statistic “*N*-len size” of contigs assembled, which has been used in previous studies [11, 16, 35]. The *N*-len, as a function of a designated length *L*, is computed as follows: the contigs are sorted in decreasing order of length, therefore the *N*-len(*L*) statistic means the shortest length of the contigs, such that the total length of the contigs with a size no shorter than *N*-len exceeds the length *L*. Since contigs with assembly errors may lead to misleadingly large *N*-len sizes, we used the correct *N*-len size to evaluate the contiguity.

Herein, we compared the assembly generated by InteMAP with the assemblies generated from five assemblers (ABySS, CABOG, IDBA-UD, SOAPdenovo, and MetaVelvet). In addition, we included Bambus 2 [35] and three recently developed assembler methods Ray [17], SPAdes [18, 19], and Omega [22] for comparison. Ray and Omega are metagenome-specific assembly methods. Bambus 2 is not a complete assembly tool, but a scaffolder to scaffold unitigs generated by other assemblers, such as CABOG or SOAPdenovo. In this comparison, we used CABOG to generate unitigs for Bambus 2. For Bambus 2, CABOG, IDBA-UD, Omega, Ray, and SPAdes, we selected their best assembly by examining both total cover length and *N*-len size of the contigs from multiple runs with different parameters. For each of ABySS, MetaVelvet and SOAPdenovo (*k*-mer graph-based), we selected two assemblies, one of which was set a small *k*-mer size and reached the best cover length, while the other was set a large *k*-mer size and reached the best contiguity.

**Fig. 3:**
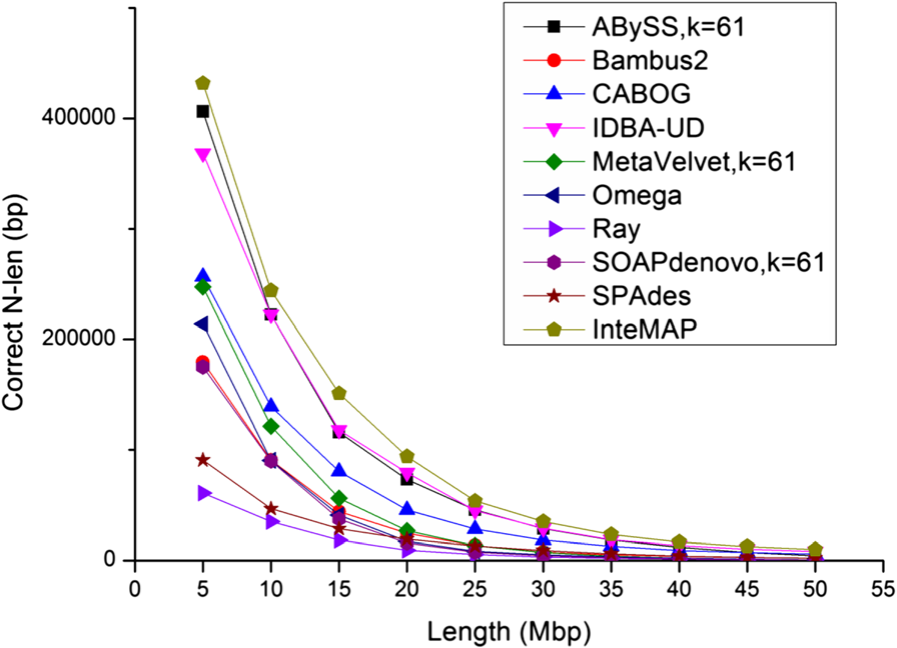
*N*-len size plot for assemblies on the *sim-113sp* dataset

The test on dataset *sim-113sp* shows that InteMAP assembly provides both the longest cover length and the best contiguity among all the compared assemblies (see Table 1). As demonstrated in the first column of Table 1, the total covered length assembled by InteMAP reaches 266.8 Mbp, while the second best is 244.8 Mbp by CABOG. We further plotted correct *N*-len size of different lengths *L* ranging from 5 Mbp to 50 Mbp. The second column of Table 1 shows that when *L* is 10 Mbp InteMAP presents the best contiguity with the longest *N*-len size of 244,190 bp than any other assembler does. More importantly, as shown in Fig. 3, InteMAP assembly always has the longest correct *N*-len size when *L* ranges from 5 Mbp to 50 Mbp. Further analysis demonstrates that InteMAP assembly has the overall best level of contiguity both in high and low coverage species (see Additional file 1: Figure S6 for more results of cover length and contiguity of assemblies generated from ABySS, MetaVelvet and SOAPdenovo with different *k*-mer sizes). Generally, the *N*-len at the lower length point represents contiguity of the sequences from higher coverage species because these are apt to generate longer contigs, while the *N*-len at the longer length point reflects contiguity of the sequences from lower coverage species. Omega assembly shows quite a lot of structure errors and has a short total contig length and *N*-len. This may be because Omega has superiority mainly in assembling a long-read dataset, but not on a short-read dataset [22].

**Table 1:** The summaries of assemblies on simulated metagenomic *sim-113sp* dataset

	Total cover length (Mbp)	Corr. *N*-len at 10 Mbp (bp)	*E*-size (bp)	Num. of covered genes	Total errors	Kbp/errors	Identity (%)
ABySS,*k* = 31	163.8	185,122	11,466	42,376	11,654	14.1	99.8
ABySS,*k* = 61	85.5	222,581	15,395	33,997	6,719	12.7	**99.9**
Bambus2	232.5	90,788	6,531	40,139	259,320	0.9	99.5
CABOG	244.8	139,195	10,142	47,968	2,482	98.6	99.8
IDBA-UD	227.9	222,631	14,651	67,713	5,416	42.1	99.7
MetaVelvet,*k* = 23	182.8	5,437	689	23,971	3,271	55.9	99.8
MetaVelvet,*k* = 61	76.3	121,245	8,628	26,747	251	304.1	**99.9**
Omega	75.8	90,383	7,751	25,837	78,078	0.9	99.8
Ray	90.2	35,059	2,365	22,958	**45**	**2005.5**	99.8
SOAPdenovo,*k* = 23	203.0	2,116	345	14,253	1,717	118.3	99.8
SOAPdenovo,*k* = 61	75.2	89,811	6,078	24,081	1,921	39.1	**99.9**
SPAdes	175.3	46,658	4,470	34,954	30,942	5.66	99.8
InteMAP	**266.8**	**244,190**	**17,652**	**70,859**	5,072	52.6	99.8

Only contigs with length ≥200 bp are considered. “*k* = 23”, “*k* = 31” and “*k* = 61” in the first column denote the assembler use the option of *k*-mer size at 23 bp, 31 bp and 61 bp. Bambus 2 uses unitigs from CABOG. Total cover length denotes the total length of reference sequences that are covered by contigs. Corr. *N*-len denotes the correct *N*-len size. *E*-size is also computed using correct contigs. Only complete covered genes are counted. Errors denote the structural errors in contigs. The error rate is measured as the average distance between errors. Identity denotes the average identity of the alignments between contigs and references, where unmapped segments of contigs are not considered. Values in bold indicate the best in the column

Furthermore, we calculate *E*-size statistic which was proposed in a previous study [34]. *E*-size is defined as the expectation of the length of the contigs that contains a position (base) randomly designated in reference genomes. This statistic addresses the question of how many genes are completely contained in the contigs. Also, it reflects both the assembly cover rate and the contiguity of the assembly. As we can see from the third column in Table 1, InteMAP has an *E*-size of 17,652 bp, the largest among all the assemblers.

Besides, we calculated the number of genes that were completely contained in the contigs of each assembly (see section Methods). The fourth column of Table 1 shows that the InteMAP assembly contains the most complete genes in contigs with the number 70,859. As one of the main goals of metagenomic assembly is to recover as many genes as possible from the sequencing reads, it is clear that the most genes covered by InteMAP demonstrate the superiority of our pipeline on metagenomic assembly.

**Fig. 4:**
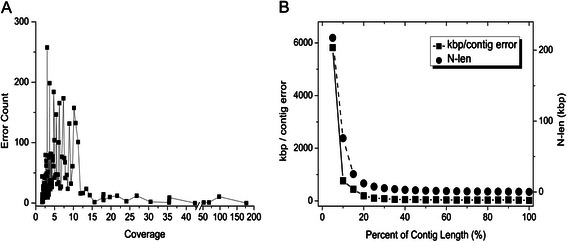
The error profile of InteMAP assembly on the sim-113sp dataset. **a** The Error counts for each species from the InteMAP assembly on the *sim-113sp* dataset are shown. The lateral axis shows the coverage of the species. **b** The square dot with solid line reflects the average error rate within the subsets of contigs with different intervals of length which were generated as follows. We sorted the contigs by the descending order of length and partitioned the set of ordered contigs into subsets so that the aggregated length of contigs in each subset equaled to or approximated to 5 % of the total length. The error rate is measured as the average distance between errors on each subset of the contigs. The error rate (left vertical axis) is plotted versus the quantile of the total length (lateral axis) at which the set of contigs are partitioned. The circle dot with dash line draws *N*-len size (right vertical axis) at the aggregate length points (percentage of the total length is shown on lateral axis)

To evaluate the accuracy of assembly, we then counted structural assembly errors and computed single-base accuracy for each assembly. For a fair comparison, we computed the error rate by calculating the ratio of total contig length to total error number, which indicates the average length of sequence containing one error. For single-base accuracy, we computed the average identity between correct contigs and the references. The total number of structural assembly errors, the length per error, and the average identity of contigs for each assembly are listed in the fifth, the sixth, and the seventh column of Table 1. The error rate of the assembly by InteMAP is lower than that by ABySS, Bambus 2, IDBA-UD, Omega, and SPAdes, while is slightly higher than that by CABOG, MetaVelvet, Ray, and SOAPdenovo. We further examined the distribution of contig errors in the InteMAP assembly. In fact, as shown in Fig. 4, most of the structural assembly errors occur in the contigs with low coverage or short length. The contigs are sorted by descending order of length. Within the top 5 % of total contig lengths, errors occur on average once every 5,821 Kbp, and this average error rate is much less frequently than that in total contigs lengths with once every 52.6 Kbp. Within the contigs mapping to the species with depth >30×, there are only 28 errors, with an average error rate of one error in every 1,014 Kbp. These results mean that the long contigs generated by InteMAP are highly reliable. This is due to InteMAP's strategy in merging contigs, which leads to effective validating contigs and correcting mis-assemblies, especially on contigs with high coverage. In low coverage sequenced species, InteMAP employs IDBA-UD to assemble the reads, which is more inclusive than other assemblers, with longer contig and more genes predicted at the cost of a higher error rate. Nonetheless, InteMAP corrects part of the errors from IDBA-UD assembly by merging assembly from CABOG in low coverage sequences. In addition, the identity between correct contigs of InteMAP and reference genomes reaches 99.8 %, which is at the same level of performance among all other assemblers with different parameters.

In conclusion, the InteMAP pipeline has an overall better performance than ABySS, Bambus 2, IDBA-UD, SPAdes, and Omega in terms of assembly cover rate, contiguity, and accuracy. Compared to CABOG, MetaVelvet, Ray, and SOAPdenovo, the InteMAP pipeline is superior on assembly cover rate and contiguity at the cost of a slightly higher error rate in short contigs or contigs from species with low sequencing coverage. It should be noted that long contigs provide more valuable genomic contents and the dominant species are often the key object to be studied in the microbial community. In this regard, compared to CABOG, MetaVelvet, Ray, and SOAPdenovo, the total superiority of the InteMAP pipeline in terms of high cover rate, high contiguity and low error in long contigs or contigs from species with high coverage indicates that InteMAP performs better than the tools CABOG, MetaVelvet, Ray, and SOAPdenovo.

### Evaluating the merging method in InteMAP

InteMAP is not a specially-designed merging algorithm, but a pipeline which detects high coverage and low coverage sequences, employs optimal assemblers to assemble different coverage sequences, and finally uses a novel merging algorithm to merge assemblies together. Nonetheless, we compared the merging algorithm in InteMAP with two specially-designed merging algorithms MeGAMerge [28] and Minimus2 [36]. Minimus2 is a part of AMOS package [36] which has been used to merge assemblies, and MeGAMerge is a method developed to merging assemblies from metagenomic data. To compare InteMAP's merging algorithm with these two tools, we used Minimus2 and MeGAMerge to replace the InteMAP merging step in the pipeline for this study only and assembled the *sim-113sp* dataset. Table 2 shows the results from three merging methods in each step of the InteMAP pipeline. In general, the results from MeGAMerge and InteMAP merging algorithm have a similar level. MeGAMerge generates longer contigs and covers more genes, while InteMAP merging generates fewer structural errors, possibly because that the InteMAP merging algorithm uses a relative conservative strategy which detects inconsistent contigs from two assemblies as potential errors and then either corrects errors or splits the contigs (see section Methods for detailed description). Minimus2 generates much smaller contigs and covers fewer genes than other two methods.

**Table 2:** Comparison of InteMAP merging algorithm with MeGAMerge and Minimus2

	Total cover length (Mbp)	Corr. *N*-len at 10 Mbp (bp)	*E*-size (bp)	Num. of covered genes	Total errors	Kbp/errors	Identity (%)
Merging high-coverage-sequence assemblies (from IDBA-UD and ABySS)							
InteMAP	202.3	145,649	9,960	12,491	50	405.6	99.9
MeGAMerge	205.9	154,674	10,290	12,649	94	219.1	99.9
Minimus2	11.9	40,393	6,630	7,237	63	188.7	99.9
Merging low-coverage-sequence assemblies (from IDBA-UD and CABOG)							
InteMAP	262.7	118,056	10,317	65,809	5,059	51.8	99.8
MeGAMerge	269.3	145,022	11,534	69,324	5,287	50.9	99.7
Minimus2	108.4	39,454	7,233	30,918	3,029	35.7	99.8
Final merged assembly							
InteMAP	266.8	244,190	17,652	70,859	5,072	52.6	99.8
MeGAMerge	273.8	253,783	18,148	72,014	5,399	50.5	99.8
Minimus2	111.3	55,893	10,285	34,892	3,045	36.4	98.8

It should be noted that the merging algorithm in InteMAP was not designed for improving any two assemblies. Indeed, merging all the assemblies from Table 1 does not results in a better assembly than the result from InteMAP pipeline (see Additional file 1: Table S1). In contrast, the assembly generated by merging all the assemblies has much more errors than the assembly generated by InteMAP pipeline, mainly because the InteMAP merging algorithm could not detect or correct all the errors contained in all of the merged assemblies. In addition, what deserves special mention is that InteMAP is more than a merging algorithm. The purpose of InteMAP is not to improve the merging algorithm, but rather provide a pipeline for a complete metagenome assembly task, which gives an optimal resolution for how to choose optimal assemblers and how to integrate assemblies step by step.

### Testing InteMAP on real NGS data

It is interesting to verify the capability of InteMAP on real metagenomic data by NGS. To this end, we selected a dataset from the 124 human gut microbial metagenomes, sequenced as a part of the MetaHIT project (sample ID: MH0012) [2]. This sample was deeply sequenced by Illumina GA machine. The high-quality paired-end reads were downloaded from NCBI Sequence Read Archive (http://www.ncbi.nlm.nih.gov/sra; ERR011117, ERR011118, ERR011119, ERR011120, ERR011121, ERR011122, ERR011123), consisting of 186 million short reads with length of 75 bp and two paired-end insert size of 134 bp and 378 bp.

We applied the InteMAP pipeline on this dataset and compared the assembly generated by InteMAP with those by other assembling tools including six metagenome-specific assemblers (Bambus 2, IDBA-UD, MetaVelvet, Omega, Ray, and SPAdes), and three general-purpose assemblers (ABySS, CABOG and SOAPdenovo). In fact, both Bambus 2 and MetaVelvet have been assessed using this benchmarking dataset in previous studies [16, 35]. However, we reran Bambus 2 and MetaVelvet on the data since we did not find the assembled data from their publications. For MetaVelvet, we selected the optimal assembly with *k*-mer size 31 from multiple runs. We reran Bambus 2 based on the unitigs generated by CABOG. As Qin et al. [2], who sequenced MH0012 sample, assembled the data using SOAPdenovo, we directly downloaded the assembly data from http://www.bork.embl.de/~arumugam/Qin_et_al_2010/ (see section Methods and Additional file 1: Supplemental Methods for detailed receipts describing how to run each assembler).

**Fig. 5:**
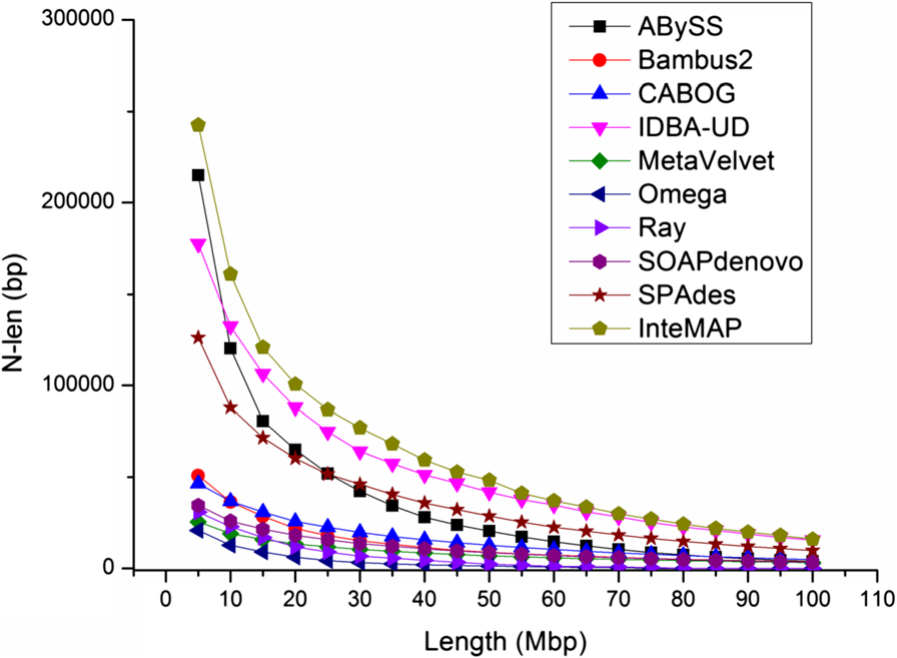
*N*-len size plot for assemblies on real NGS dataset (sample MH0012)

We then assess each assembly by computing the statistics of total contig lengths, *N*-len, *E*-size, and the number of ORFs predicted by MetaGeneMark [6] (see Table 3). As we may see from the test, the InteMAP assembly has the longest total contig lengths of 278.6 Mbp among all the assemblies. Moreover, the *N*-len size of the InteMAP assembly is much higher than those of other individual assemblies, demonstrating a significant improvement in contig contiguity (see Fig. 5). The *E*-size of the assembly generated by the InteMAP pipeline is also the largest among all the assemblies. In addition, the InteMAP assembly contains the most non-redundant ORFs and complete ORFs. Lacking reliable references, we are not able to evaluate the accuracy of the assemblies. Nevertheless, the results of the real NGS data is consistent with those from the simulated dataset, demonstrating that the InteMAP pipeline generates better assembly that has a longer total contig length, higher contiguity and contains more genes than all other individual assemblers.

**Table 3:** Assembly statistics and predicted gene number on human gut microbial metagenome dataset (Sample MH0012)

	Sum of contig length (Mbp)	*N*-len at 5 Mbp (bp)	*N*-len at 50 Mbp (bp)	*E*-size (bp)	Non-redundant ORFs predicted	Num. of predicted complete ORFs
ABySS, *k* = 61^a^	158.7	215,125	20,469	18,366	184,441	112,237
Bambus2	226.6	50,905	8,903	6,427	336,604	184,683
CABOG	185.6	46,459	12,738	6,964	222,638	119,907
IDBA-UD	277.2	177,468	41,831	23,970	339,336	186,427
MetaVelvet, *k* = 31	233.9	25,531	6,930	4,198	337,677	140,312
Omega	75.6	20,874	1,255	1,624	99,648	39,218
Ray	108.2	22,999	2,356	2,001	152,339	138,225
SOAPdenovo (Qin et al. 2010)^b^	237.4	34,518	8,679	5,166	306,657	135,644
SPAdes	181.3	126,295	28,707	16,261	190,744	149,665
InteMAP	**278.6**	**242,608**	**48,303**	**39,156**	**339,598**	**186,997**

Only contigs ≥500 bp are considered. Bambus 2 uses unitigs from CABOG. “*k* = 31” denotes that MetaVelvet uses option of *k*-mer size 31. The assembly generated by Qin et al. (2010) is included, which is assembled by SOAPdenovo. In the column of non-redundant ORFs predicted, only ORFs ≥100 bp are counted. Last column lists the number of complete ORFs. The ORFs are predicted by MetaGeneMark [6]. Values in bold indicate the best in the column

^**a**^Assembly by ABySS was generated from the correct reads from which many low coverage reads may be excluded because we failed ran ABySS on the mixed reads

^**b**^Assembly by SOAPdenovo was directly downloaded from the publication of [2]

We further tested the performance of InteMAP on two more real metagenomic data downloaded from NCBI Sequence Read Archive (http://www.ncbi.nlm.nih.gov/sra; SRR512043 and SRR413680). SRR512043 is from one of samples from the permafrost metagenome project [14], and has a totally low coverage depth, while SRR413680 is from another gut microbiota metagenome project [37] and has a similar depth with the MH0012 sample. We applied InteMAP on these two data sets and compared the results to the assemblies generated by IDBA-UD. Here we compared the InteMAP with IDBA-UD only because IDBA-UD has the totally best performance among all the individual assemblers in our previous tests. Table 4 shows that on the data SRR512043, InteMAP has totally better performance than IDBA-UD with much longer total contig lengths, longer *N*-len at 100 Kbp, and more predicted non-redundant ORFs. Notably, InteMAP only employed IDBA-UD and CABOG assemblers working on this data and merged their assemblies because there are few high coverage sequences in this data. This suggests CABOG assembled many contigs that were missed by IDBA-UD, and InteMAP merged them together and made a large improvement over IDBA-UD. On the dataset SRR413680, the InteMAP assembly has slightly longer total contigs and more predicted ORFs. The evident longer *N*-len at 10 Mbp of InteMAP assembly than IDBA-UD assembly indicates InteMAP improves largely in the contiguity of the assembly over IDBA-UD.

**Table 4:** Comparison of IDBA-UD and InteMAP on two real metagenomic data samples

	IDBA-UD	InteMAP
*SRR512043*		
Sum of contig length (bp)	6,545,817	17,134,336
*N*-len at 100 Kbp (bp)	607	1,355
Non-redundant ORFs predicted	16,767	53,542
*SRR413680*		
Sum of contig length (bp)	147,576,380	149,997,510
*N*-len at 10 Mbp (bp)	112,393	205,465
Non-redundant ORFs predicted	163,430	167,133

The InteMAP pipeline is designed for convenient use with automatic one-step operation; meanwhile users can also run the pipeline step by step, so that they can get an optimized outcome from multiple runs of different parameters for each individual assembler in the middle process. As consisted of several assembly processes by individual assemblers and mapping processes by Bowtie2, it takes longer to run InteMAP than other individual assemblers. For instance, in our test on the dataset MH0012, it took us for about 3 days to finish running InteMAP on the machine with the memory of 192G and thread number of 22, while it took for within one day to run individual assemblers on the same machine.

## Discussion and conclusions

It has been believed that the assembly for metagenomic NGS reads has not reached a point to recover one or more complete genomes with the exception of finishable dominant species [23]. However, it is never a trivial matter to make efforts to develop strong and efficient assembly tools to provide more valuable genomic content beyond raw data with a large number of NGS reads, even researchers can directly obtain a little information based on these raw data or 16S sequences [1]. In this work, we have performed a comprehensive assessment on state-of-the-art *de novo* assemblers to understand their advantages and disadvantages in assembling metagenomic reads. The analysis revealed that the assembly performance of individual tool critically depends on sequence coverage and a combinatorial application of different tools is desired to achieve an overall better performance to assemble short reads sequenced with uneven coverage as in a real metagenomic sample. The InteMAP pipeline addressed this question by integrating three leading assemblers, IDBA-UD, ABySS, and CABOG, to assemble metagenomic NGS reads. The essential manipulation of InteMAP is to decide the assembling approaches according to sequencing coverage estimated for each short read. Thus, the pipeline actually presents a smart platform suitable to assembling real metagenomic NGS data with uneven coverage distribution of sequencing depth. Our analysis showed that the estimated coverage for each read does not exactly mean its real coverage of sequencing, which is no more than calculated based on the draft assemblies by IDBA-UD and ABySS. However, the estimation plays an important role in the pipeline, moreover it leads to the fact that InteMAP achieves a high performance in assembling metagenomic NGS reads.

Surprisingly, several newly developed methods for metagenomic assembly such as Omega [22], and Ray [17] do not work well for our datasets. Ray has surprising low performance in terms of contig size and covered genes on the tested datasets, but has an extremely low error rate, suggesting Ray is a quite conservative assembler which tends to avoid errors while generating fewer contigs. Omega employs a minimal cost flow analysis on an overlap graph to generate unitigs. However, unlike CABOG which allows mismatch in the overlap detection, Omega uses exact-match overlaps to construct overlap graph, making it highly sensitivity to the sequencing errors or SNPs. Thus, Omega does not deal well with a highly complex metagenome dataset which might contain a large amount of low-sequencing-depth species on which sequencing errors could not get corrected easily.

The assembler space is constantly moving and more assemblers are developed. It will be interesting to exam new assemblers to find their particular strength in the metagenomic assembly and further plug them in the InteMAP pipeline. However, as we have shown, merging all the assemblies did not result in a better assembly than what InteMAP did, arbitrary merge will not improve the assembly, unless we know the respective strength of an assembler and integrate it in an appropriate way.

Metagenomes are complex mixtures usually containing diverse species with different abundances. An ideal metagenomic assembler is expected to perform well on both high and low depth sequences within the metagenomes. On the condition of a certain sequencing capacity, high depth sequences usually represent dominant or high-abundance species within the community. Conversely, low-coverage sequences usually represent low-abundance species which often occupy a large proportion of the community, especially in high-complexity metagenomes, such as the ocean and the soil metagenomes [1, 38]. In metagenomes which have dominant species, to construct the high-quality draft assemblies or even complete genomes of dominant species has a great significance in metagenomic studies [39]. On the other hand, to assemble as many low abundant sequences as possible is also very important for the completeness of metagenomic studies. By integrating different assemblers which are good at assembling sequences at different coverage levels, the InteMAP pipeline demonstrates strong capability in assembling both high and low depth sequences, based on the improvements in terms of contiguity and total cover length, in comparison with current leading assemblers on both simulated and real NGS data.

## Methods

### Simulated dataset

To construct the simulated metagenome dataset, we used the MetaSim simulator [40] to generate a collection of synthetic metagenomic reads which reflect the specified abundance distribution of the species. In the current study, we mainly target the Illumina reads although our pipeline is also able to be applied on other short reads. To imitate Illumina sequencing, the read length was set 100 bp, with the average and the standard deviation of paired-end insert size as 300 bp and 20 bp. To model the specific pattern of sequencing error of Illumina technology, we used NGSfy [41] to generate sequencing errors in reads, which uses a fourth-degree polynomial model to describe the frequency of errors in Illumina reads. We used the default settings for NGSfy, and the average error rate was 1.5 %, as in previous studies [41, 42].

We constructed the *sim-113sp* dataset by mixing the synthetic reads from 113 complete microbial genomes downloaded from NCBI RefSeq database. The collection of the 113 species is exactly the same as used by Mavromatis et al. [43] to generate simulated metagenomic datasets, except for a few unfinished genomes, which we chose close relatives (usually a different strain) instead (see Additional file 2: File S1). For the abundance setting, we used the logarithmic distribution, which has been used in Mende et al. [30] to model the microbial abundance distribution. The highest sequencing coverage of species in this dataset is 177.5×, followed by 97.6×, and the coverage drops to below 10× after the top 20 species (see Additional file 1: Figure S7). We totally generated 4 million reads of totally 400 Mbp, which are able to be downloaded from http://cqb.pku.edu.cn/ZhuLab/InteMAP/index.html.

In order to calculate the number of genes contained by the assembly in the evaluation on simulated metagenomic dataset *sim-113sp*, we downloaded the gene annotation files for each reference genome, which record the start and ending positions for each gene on the genome sequence, from the NCBI database. According to the mappings between the contigs and the reference genomes, if one gene was completely contained in an aligned region of the references, this gene was deemed completely contained by the contigs.

### Running the assemblers

The pre-processing of correcting the sequencing errors in raw reads has been reported to substantially enlarge the contig size for some assemblers in the single genome assembly [34]. In the current study, error correction also improved the metagenome-assembly quality, especially on the high-depth species. The InteMAP pipeline employs a module of error correction to pre-process the reads before assembling. For a fair comparison, in our experiments, reads were pre-processed by the error correction process before being assembled by all assemblers (see Additional file 1: Supplemental Methods for the detailed process of error correction).

For each assembler, we ran multiple times with different parameter settings and selected the ones that appeared optimal or near optimal results (the parameters selected and details describing how to run each of the assembler are in Additional file 1: Supplemental Methods). The contigs by ABySS, IDBA-UD, MetaVelvet and SOAPdenovo were extracted from their scaffold sequences after splitting the scaffold sequences at gaps. SOAPdenovo has an additional module named GapCloser as a post-processor. Our test demonstrated that the GapCloser's post-processing actually increases the assembling errors dramatically. Therefore, we selected the assemblies without the post-processing of GapCloser for SOAPdenovo.

### Evaluating the assemblies

Because extremely short contigs, in many cases as small as the *k*-mer size used to build the *k*-mer graph, can hardly support information for further analysis, for all the assemblies, contigs with length <200 bp were excluded before evaluation, as did in previous study [34]. Following the way used in the previous study [34], we used MUMmer package v3.23 [44] to evaluate the contigs, in which *nucmer* [44] was used to align the contigs against the references with the options “-maxmatch -l 30 -banded -D 5.” Then *delta-filter* with the option “-i 95 -o 95” and *dnadiff* [45] were used to obtain a globally optimal mappings between contigs and references, from which we could directly get the structural assembly errors (segmental indels (>5 bp) and misjoins of two non-adjacent segments). From the output of *dnadiff*, we also got the identity of the aligned contigs. In the evaluation of the assemblies on simulated dataset, we used corrected contigs to evaluate the contiguity of the contigs by computing the correct *N*50 size, correct *N*-len size, and correct *E*-size. We extracted the aligned contig fragments as the corrected contigs from the mappings between contigs and references generated by *dnadiff* (aligned fragments end at the ends of contigs or assembly errors).

The *N*-len size is computed as defined in the RESULTS section. Herein it is should be noted that *N*50 size corresponds to the *N* -len(*x*) for *x* = *L/*2, where *L* denotes the reference genome length. The computation of the *E*-value follows the formula

$$ E={\displaystyle \sum_C\frac{{L_C}^2}{G},} $$

where *L*_*C*_ is the length of contig *C*, and *G* is the total reference genome length. On real NGS dataset MH0012, as the total length of reference genomes is not available, the *G* in the *E*-size formula was assigned the maximal total contigs length among all the assemblies run on this dataset (we used 280,000,000 here).

### Algorithm of the InteMAP pipeline

**Fig. 6:**
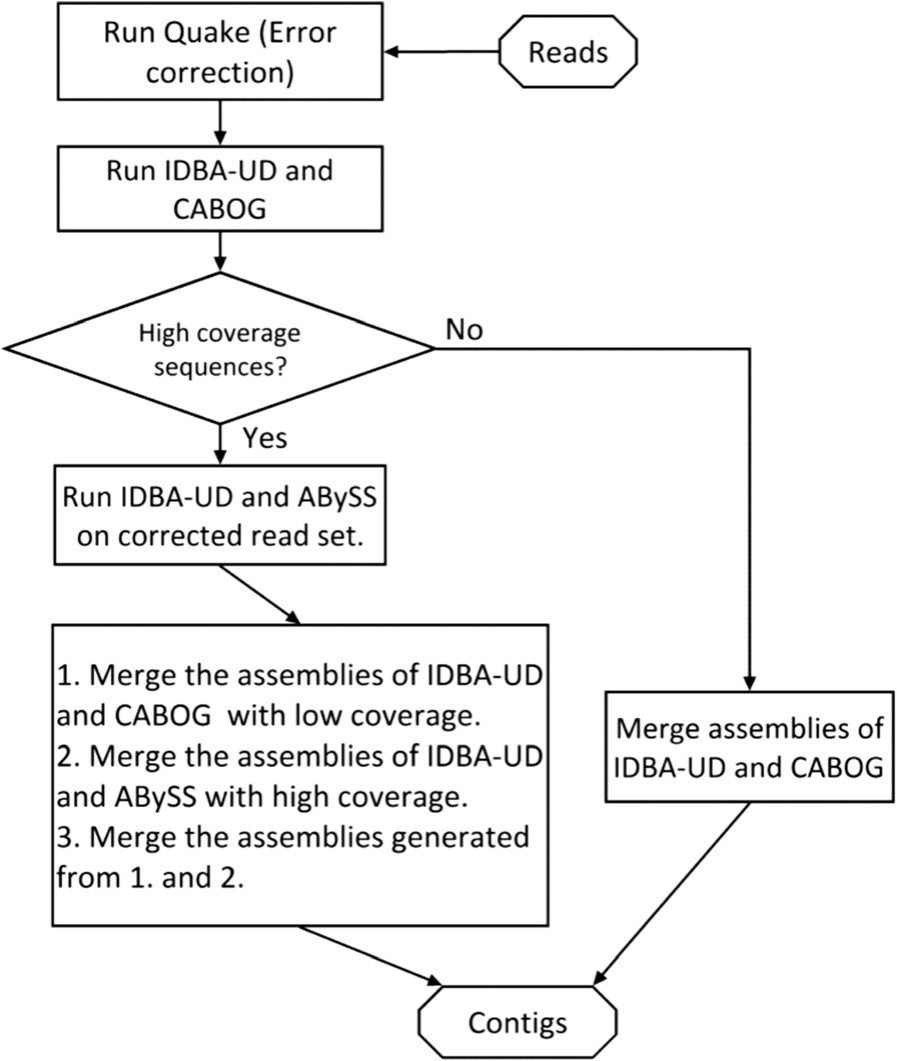
The flowchart of the main procedures of the InteMAP pipeline

We then describe the procedures of the InteMAP pipeline step by step as follows (see Fig. 6):

- *Step 1. Correcting errors of the input reads.* By default, input reads are first pre-processed by the Quake tool [46], which aims to correct the sequencing errors in high-coverage sequences (see Additional file 1: Supplemental Methods for details). Two sets of reads are then generated: one named “correct read set” consisting of reads corrected or validated by Quake, and the other consisting of the remaining ones (see Additional file 1: Figure S8 for the ratio of the correct reads to total reads for each coverage of species). Two sets are mixed together and the mixed read set is termed “total read set”.
- *Step 2. Running IDBA-UD and estimating reads coverage.* After the assembly by IDBA-UD, reads are mapped into the contigs using the read alignment tool, Bowtie 2 [47]. According to the mappings between reads and contigs, the coverage of each read is then estimated by the times of the total length of reads to the length of the assembled contig. Furthermore, the contigs are separated along with the mapped reads by different coverage. The pipeline then picks up both the reads mapping to contigs with the coverage < *L* (this can be designated by users, 50× by default) and the unmapped reads, and assembles them using CABOG.
- *Step 3. Checking the total length of high coverage contigs.* The pipeline first checks the total length of the contigs with the coverage >30×. If the length is shorter than a designated value (*e.g.* 1 Mbp by default), the pipeline goes to Step 6 for the condition of the low-coverage reads assembly. Otherwise, goes to the next step.
- *Step 4. Assembling reads of the “correct read set” using ABySS and IDBA-UD respectively.* The “correct read set” has been generated in Step 1, and using this set can largely increase the quality of assembly for the high-depth species by both ABySS and IDBA-UD. After assemblies are generated by ABySS and IDBA-UD, the pipeline maps the reads into the contigs and picks up the high-depth contigs for both assemblies. The minimal coverage of high-depth contigs for ABySS and IDBA-UD is set 20× by default.
- *Step 5. Merging the assemblies at high and low coverage levels iteratively.* First, the pipeline merges the high-depth contigs from the IDBA-UD and ABySS assemblies generated in Step 4, using the algorithm described below. Then filter out the high-coverage contigs (≥50×) by IDBA-UD in Step 2 and merge the remaining contigs by CABOG in Step 2. Since the pipeline has merged contigs both on the high and low coverage sequences, finally, the pipeline merges them together as the final contigs. Note that the ranges of the high and low coverage overlap each other to ensure that the pipeline covers all the sequences, so the default cut-off for high-depth and low-depth are set to make the range broader than those used for the assembly evaluation. The pipeline ends at this step.
- *Step 6. Merging the assemblies by IDBA-UD and CABOG.* If Step 4 and 5 are skipped, which means that there are no high coverage species within the metagenomes, merge the contigs generated by both IDBA-UD and CABOG in Step 2 as the final contigs. The pipeline ends at this step.

### The algorithm for merging two sets of contigs

The steps of merging contigs in the InteMAP pipeline are described as follows.

**Fig. 7:**
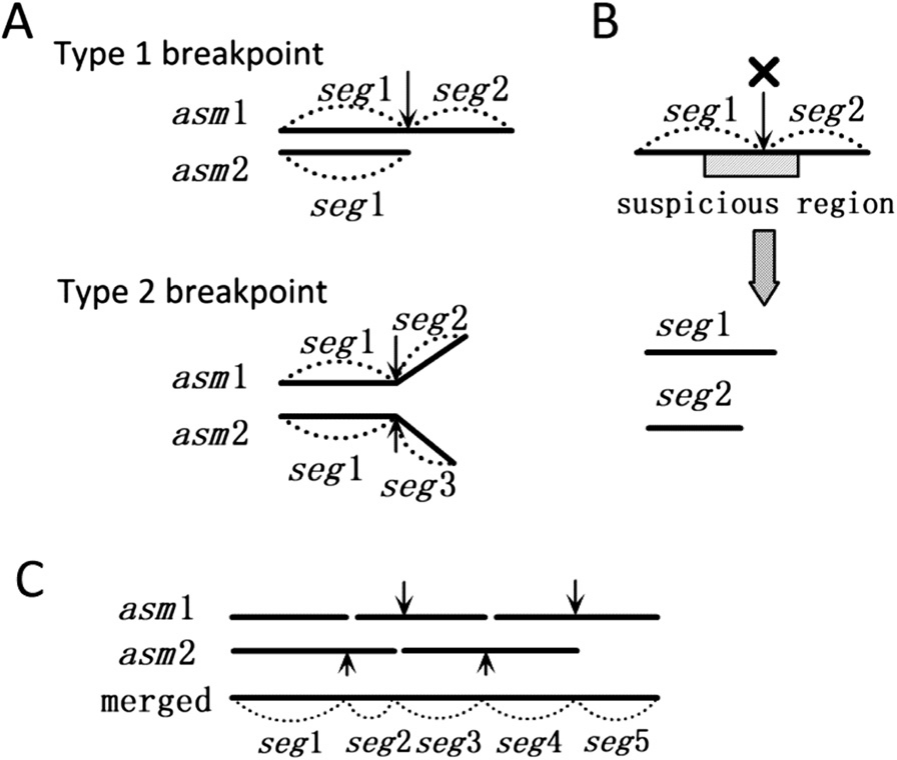
Illustration of merging two assemblies. **a** Two types of breakpoints caused by differences between two assemblies are illustrated. Suppose two assemblies *asm*1 and *asm*2 have the same assembled sequence segment *seg*1. The first type of difference is that in *asm*1, *seg*1 is extended with *seg*2 on the right, while in *asm*1, *seg*1 ends on the right. The second type of difference is that in *ass*1, *seg*1 is extended with *seg*2 on the right, while in *asm*2, *seg*1 is extended with *seg*3 on the right. **b** Split the contigs at the breakpoint within the suspicious region. **c** An example of merging the segments at the breakpoints which are not broken
- *Step 2. Validating the contigs.* The contigs are validated by mapping the paired-end reads against the contigs, which has been used in previous work [45]. The basic idea is that if one contig is correctly assembled, each pair of paired-end reads is located and mated correctly. If a region has ≥5 mis-mated reads located, this region will be marked as suspicious which indicates potential mis-assembly within the region. Thus, if one breakpoint is located within the suspicious region, it is more likely to be an assembly error. Therefore, the algorithm breaks all the breakpoints which are located within the suspicious regions (Fig. 7b).
- *Step 3. Breaking the contradictory breakpoints.* The remaining breakpoints are treated differently according to their types. As Type 1 breakpoints are mated and in different assemblies respectively, if both mated breakpoints are not broken in Step 2, both of them will be broken at this step. In contrast, if only one Type 1 breakpoint is broken in Step 2, the other breakpoint will automatically transform to the Type 2 breakpoint. The survived Type 2 breakpoints are reserved.
- *Step 4. Connecting the segments at reserved breakpoints* (Fig. 7c). Because there are no conflicting breakpoints remained after Step 2 and 3, each segment has at most one segment linked by the reserved breakpoint in either the left or the right direction. Then, beginning from any segment which ends at one side, the pipeline extends the segment by connecting the adjacent one until the extending segment ends at the extending direction. The final contigs are generated by reading the bases from the merged segments. If the segment is shared by two assemblies, randomly select one strand of sequences to read the bases.

- *Step 1. Comparing the two sets of contigs.* It begins with comparing the two sets of contigs using *nucmer*, *delta-filter*, and *dnadiff* in MUMmer package to get the mappings between the two sets of contigs. From the mappings, the same segments shared by two sets of contigs are marked. Then, the breakpoints are marked inside the contigs where the bifurcations appear (Type 1 breakpoint) or the segments end in the contigs of the different assembly (Type 2 breakpoint), as illustrated in Fig. 7a. The breakpoints may indicate assembly errors.

## Availability of supporting data

The package of the InteMAP software and all the benchmarking data are freely available from http://cqb.pku.edu.cn/ZhuLab/InteMAP/index.html.

## Additional files

Additional file 1:
**Supplemental Materials.** This file is in docx format and includes the Supplemental Methods and Supplemental Figures. (DOCX 784 kb)
Additional file 2:
**Supplemental file 1.** This file is in xls format and records the information of the 113 species selected to generate the dataset *sim-113sp. (XLS 26 kb)*
