# Supplementary material for: InteMAP: Integrated metagenomic assembly pipeline for NGS short reads
Source: BMC Bioinformatics. 2015 Aug 7;16:244. doi: 10.1186/s12859-015-0686-x (PMC4545859; doi:10.1186/s12859-015-0686-x)

**Supplemental Files**

**Supplemental File 1** is a separate file in .xls format, which lists the basic information for each species including name, GI, genome length and total read length, in the simulated *sim-113sp* dataset.

**Supplemental Methods**

**Evaluating the impact of the mixture of different species to the metagenomic assembly problem**

To assess how the mixture of different species impacts assemblers in metagenomic assembly, we constructed four datasets, named *sim1*, *sim2*, *sim3,* and *sim4* (Supplemental Table 1). Each dataset is comprised of three species. To see how uneven-abundance distribution affects the metagenomic assembly, *sim1* and *sim2* were constructed on the same species, but with even- and uneven- abundance distributions respectively. Similarly, *sim3* and *sim4* had the same species, and the abundance settings were the same as those of *sim1* and *sim2*. However, the species of *sim1* and *sim2* were far relatives (different genus) while those of *sim3* and *sim4* were close relatives (same genus). For each assembler and each dataset, we ran assemblers first on the mixture of the reads, and then on the separate sets of reads from individual species respectively. The differences between the assemblies from the mixture of reads (mixed assembly) and from separate sets of reads (separate assembly) reflected the extent of the impact from the mixture of different species. For almost all the assemblers, the mixture of the uneven-coverage species did not result in a significant variance in correct *N*50 size or error count statistic between the mixed assembly and separate assembly, in contrast to the result from the mixture of the even-coverage species, (Supplemental Figure 6 and 7). The only remarkable impact of the uneven coverage was the drastically increase in the assembly errors from ABySS. On the other hand, the mixture of the closely related species had a significant impact on both correct *N*50 size and error count statistic, in contrast to the result from the mixture of far related species (Supplemental Figure 6 and 7). From this result, the impact from the mixture of different species seemed universal for all assemblers in our experiments.

| **Table.** Details of four simulated datasets. | | |
| --- | --- | --- |
|  | Species | Coverage |
| *Sim1* | *Staphylococcus aureus* | 30× |
|  | *Streptococcus parasanguinis* | 30× |
|  | *Gardnerella vaginalis* | 30× |
| *Sim2* | *Staphylococcus aureus* | 10× |
|  | *Streptococcus parasanguinis* | 30× |
|  | *Gardnerella* *vaginalis* | 50× |
| *Sim3* | *Chlorobium limicola* | 30× |
|  | *Chlorobium phaeobacteroides* | 30× |
|  | *Chlorobium phaeovibrioides* | 30× |
| *Sim4* | *Chlorobium limicola* | 10× |
|  | *Chlorobium phaeobacteroides* | 30× |
|  | *Chlorobium phaeovibrioides* | 50× |

**Error correction**

Error correction is built in the InteMAP pipeline, and we also preprocessed the reads by error correction before using other algorithms to assemble them. Without any appropriate tool specially for metagenomic error correction at present, we adopted Quake (Kelley et al. 2010) for metagenomic data by setting a constant cutoff of *k* (we set 1 here), above which the times of the *k*-mer occurrences were deemed necessary to trust that *k*-mer. Taking the *sim-113sp* dataset as an example, we run Quake using the receipt as follows:

> jellyfish count -q --quality-start 64 -c 8 -o sim-113sp.db -m 17 -t 12 -s 2G sim-113sp_1.fastq sim-113sp_2.fastq

> mv sim-113sp.db_0 sim-113sp.dbm

> jellyfish qdump -c sim-113sp.dbm > sim-113sp.qcts

> echo “sim-113sp_1.fastq sim-113sp_2.fastq” > sim-113sp-files

> correct -f sim-113sp-files -k 17 -m sim-113sp.qcts -c 1 -q 64 -u -p 12 --log

We mapped the correct reads (corrected and confirmed correct by Quake) from *sim-113sp* dataset back to the reference genomes and found that the error rate dropped to about 0.5% from its original value 1.5%. However, the error correction is only effective for the high-coverage species. For the low-coverage species, most reads remained uncorrected (Supplemental Fig. 9), so we mixed the correct reads and uncorrected reads together, and used these mixed reads as our benchmarking data, so that assemblers can also work on reads for the low-coverage species. We have uploaded the data of original, correct and mixed reads at <http://cqb.pku.edu.cn/ZhuLab/InteMAP/sim-113sp.html>.

**MH0012 data**

The raw data downloaded consist of seven sets of reads (ERR011117-ERR011123), where ERR011117, ERR011118, ERR011120, ERR011121 use a short insert size with an average length at 128 bp, and ERR011119, ERR011122, ERR011123 use a long insert size with an average length at 355 bp. We merged the seven read sets into two sets by their insert size, named *libshort* and *liblong*. Like the sim-113sp dataset, the *libshort* reads and the *liblong* reads are both processed by error correction. The original reads and the processed reads are available at <http://cqb.pku.edu.cn/ZhuLab/InteMAP/MH0012.html> .

**Recipts for running assemblers**

**ABySS.** The version of ABySS we used is version 1.2.7. Suppose the *k*-mer size we used is $***k***, we ran ABySS by the following command:

For sim-113sp:

> abyss-pe k=$***k*** n=5 name=asm in=”sim-113sp-mix_1.fastq sim-113sp-mix_2.fastq”

For MH0012:

> abyss-pe k=$***k*** n=5 name=asm lib=”libshort liblong” libshort=”libshort- cor_1.fastq libshort- cor_2.fastq” liblong=”liblong-cor_1.fastq liblong-cor_2.fastq”

**CABOG.** The version of CABOG we used is release 7.0. We ran CABOG using this command:

For *sim-113sp*:

**>** fastqToCA -insertsize 300 20 -libraryname sim-113sp -type illumina -mates sim-113sp-mix_1.fastq,sim-113sp-mix_2.fastq > sim-113sp.frg

**>** runCA -d . -p asm -s config.spec sim-113sp.frg

For MH0012:

> fastqToCA -insertsize 128 10 -libraryname short -mates libshort-mix_1.fastq,libshort-mix_2.fastq > libshort.frg

> fastqToCA -insertsize 355 15 -libraryname long -mates liblong-mix_1.fastq,liblong-mix_2.fastq > liblong.frg

> runCA -d . -p asm -s config.spec *.frg

The parameters specified in config.spec are as below:

utgErrorRate=0.12, ovlErrorRate=0.14, cnsErrorRate=0.14, cgwErrorRate=0.14,

doOverlapBsedTrimming=0, merSize=14, overlapper=ovl, ovlMinLen=23,

frgCorrBatchSize=199995, ovlCorrBatchSize=199995, doExtendClearRanges=1,

unitigger=bogart, doToggle=1.

**Bambus 2.** The version of Bambus 2 we used is release 3.0.1 (in the package of AMOS 3.0.1). We used Bambus 2 to scaffold the unitigs generated by CABOG as below. Suppose the output directory of CABOG assembly is $CAdir.

> ln -s $CAdir/asm.asm .

> ln -s $CAdir/asm.gkpStore

> gatekeeper -dumpfrg -allreads asm.gkpStore > asm.frg

> toAmos -f asm.frg -o asm.afg

> bank-transact -c -b asm.bnk -m asm.afg

> toAmos_new -a asm.asm -U -b asm.bnk

> goBambus2 asm.bnk asm clk bundle reps,”-noPathRepeats” orient,”-maxOverlap 500 -rundundancy 0” 2fasta printscaff

**IDBA-UD.** The version of IDBA-UD we used is idba-1.1.0. We first transformed the .fastq files into .fasta files since IDBA-UD only support read files in fasta format. Then we handled as the command:

For *sim-113sp*:

> idba_ud -r sim-113sp-mix.fasta --mink 23 -num_threads 22 --pre_correction -o asm

For MH0012:

> idba_ud --read libshort.fasta --read_level_2 liblong.fasta --mink 23 --num_threads 22 --pre_correction -o asm

**MetaVelvet.** The version of MetaVelvet we used is the release 1.2.01. The operation of MetaVelvet requires Velvet (the version we used is release 1.2.07). Assume the *k*-mer size is $***k***, the command is:

For *sim-113sp*:

> velveth asm $***k*** -fastq -shortPaired -separate sim-113sp-mix_1.fastq sim-113sp-mix_2.fastq

> velvetg asm -exp_cov auto -ins_length 300 -ins_length_sd 20

> meta-velvetg asm -exp_cov auto -ins_length 300 -ins_length_sd 20 -scaffolding yes

For MH0012:

> velveth asm $***k*** -shortPaired -fastq -separate libshort-mix_1.fastq libshort-mix_2.fastq -shortPaired2 -fastq -separate liblong-mix_1.fastq liblong-mix_2.fastq

> velvetg asm -exp_cov auto -ins_length 128 -ins_length_sd 10 -ins_length2 355 -ins_length2_sd 15

> meta-velvetg asm -exp_cov auto -ins_length 128 -ins_length_sd 10 -ins_length2 355 -ins_length2_sd 15 -scaffolding yes

**Omega.** The version of Omega is the release 1.0.2.We used the default parameters to run Omega:

For *sim-113sp*:

> Omega -pe sim-113sp-mix_1.fastq,sim-113sp-mix_2.fastq -l 60

For MH0012:

> cat libshort-mix_1.fastq liblong-mix_1.fastq > merged_1.fastq

> cat libshort-mix_2.fastq liblong-mix_2.fastq > merged_2.fastq

> Omega -pe merged_1.fastq,merged_2.fastq -l 60

**SOAPdenovo.** The version we used is SOPAdenovo version 1.05. Assume the *k*-mer size is $***k***, we went as follows:

For *sim-113sp*:

**>﷒**echo “[LIB]\n avg_ins=300\n reverse_seq=0\n asm_flags=3\n rank=1\n q1=sim-113sp-mix_1.fastq\n q2=sim-113sp-mix_2.fastq\n” > config

> SOAPdenovo-63mer all -K $***k*** -p 12 -s config -o asm

> GapCloser -b config -a asm.scafSeq -o asm-GC.scafSeq -t 12 -p $***k***

The assembly on MH0012 by SOAPdenovo was directly downloaded from <http://www.bork.embl.de/~arumugam/Qin_et_al_2010/>, which had been assembled by Qin et al. (2010).

**SPAdes.** Version 3.3.1 is used.

For *sim-113sp:*

> python spades.py --sc --pe1-1 p113species_mix-1.fastq --pe1-2 p113species_mix-2.fastq --pe1-fr --careful -t 10 -m 150 -o SPAdes_out

For MH0012:

> python spades.py --sc --only-assembler --pe1-1 liblong_mix_1.fastq --pe1-2 liblong_mix_2.fastq --pe2-1 libshort_mix_1.fastq --pe2-2 libshort_mix_2.fastq --pe1-fr --pe2-fr --careful -m 200 -o SPAdes_out

**Ray.** Version 2.3.1 is used. Assume the *k*-mer size is $***k***.

For sim-113sp:

> mpiexec -n 8 Ray Meta -detect-sequence-files p113species/ -o Ray_out -k $***k***

For MH0012:

> mpiexec -n 8 Ray Meta -detect-sequence-files MH0012/ -o Ray_out -k $***k***

**InteMAP.** The current version of InteMAP is v1.0. InteMAP provides automatic one-step operation to process the data. We ran InteMAP using default parameters on both simulated and real data. The pipeline requires ABySS 1.2.7, CABOG 7.0, IDBA-UD 1.1.0, Quake and MUMmer 3.2.3. Suppose you have installed InteMAP package along with the necessary software and they are available in your path. Assume $dir is the path of InteMAP package.

For *sim-113sp*:

> python $dir/runInteMAP.py sim-113sp-ori-file sim-113sp-info sim-113sp-spec

For MH0012:

> python $dir/runInteMAP.py MH0012-ori-file MH0012-info MH0012-spec

The files “sim-113sp-ori-file”, “sim-113sp-info”, and “sim-113sp-spec” are available at <http://cqb.pku.edu.cn/ZhuLab/InteMAP/sim-113sp.html>. “MH0012-ori-file”, “MH0012-info”, and “MH0012-spec” are available at <http://cqb.pku.edu.cn/ZhuLab/InteMAP/MH0012.html>.

**Supplemental Tables**

**Supplemental Table 1.** Comparison of InteMAP assembly to the assembly generated by merging all the assemblies by InteMAP merging algorithm on the simulated sim-113sp dataset.

|  | Total cover length (Mbp) | | Corr. *N*-len at 10 Mbp (bp) | *E*-size (bp) | Num. of covered genes | Total errors | Kbp / errors | Identity (%) |
| --- | --- | --- | --- | --- | --- | --- | --- | --- |
| Merging all assemblies^*^ | | 265.9 | 206,431 | 14,476 | 59,500 | 162,596 | 1.6 | 99.7 |
| InteMAP pipeline | **266.8** | | **244,190** | **17,652** | **70,859** | 5,072 | 52.6 | 99.8 |

^*^12 assemblies from Table 1 in the main context generated by each individual assembler were added into merge iteratively using InteMAP merging algorithm.

**Supplemental Figures**

**Supplemental Figure 1.** Trade-off between correct contig *N*50 and error rate for each assembly from the *sim-113sp* dataset at high and medium coverage level.





Average correct contig *N*50 size versus error rates. Both are averaged over all separate assemblies from high coverage (>30×) level (A) along with medium coverage (15-30×) level (B). Error rates are measured as the average distance between errors, in kilobases.

**Supplemental Figure 2.** The distribution of the total genes on species with low coverage (<18×) covered by assemblies of ABySS, IDBA-UD, MetaVelvet, and SOAPdenovo.





The cyan part represents the genes shared by at least two assemblies. Other parts represent genes exclusively covered by ABySS (*black*), IDBA-UD (*red*), MetaVelvet (*green*) and SOAPdenovo (*blue*), respectively.

**Supplementary Figure 3.** Genes Covered by assemblies for high coverage (>18×) species.


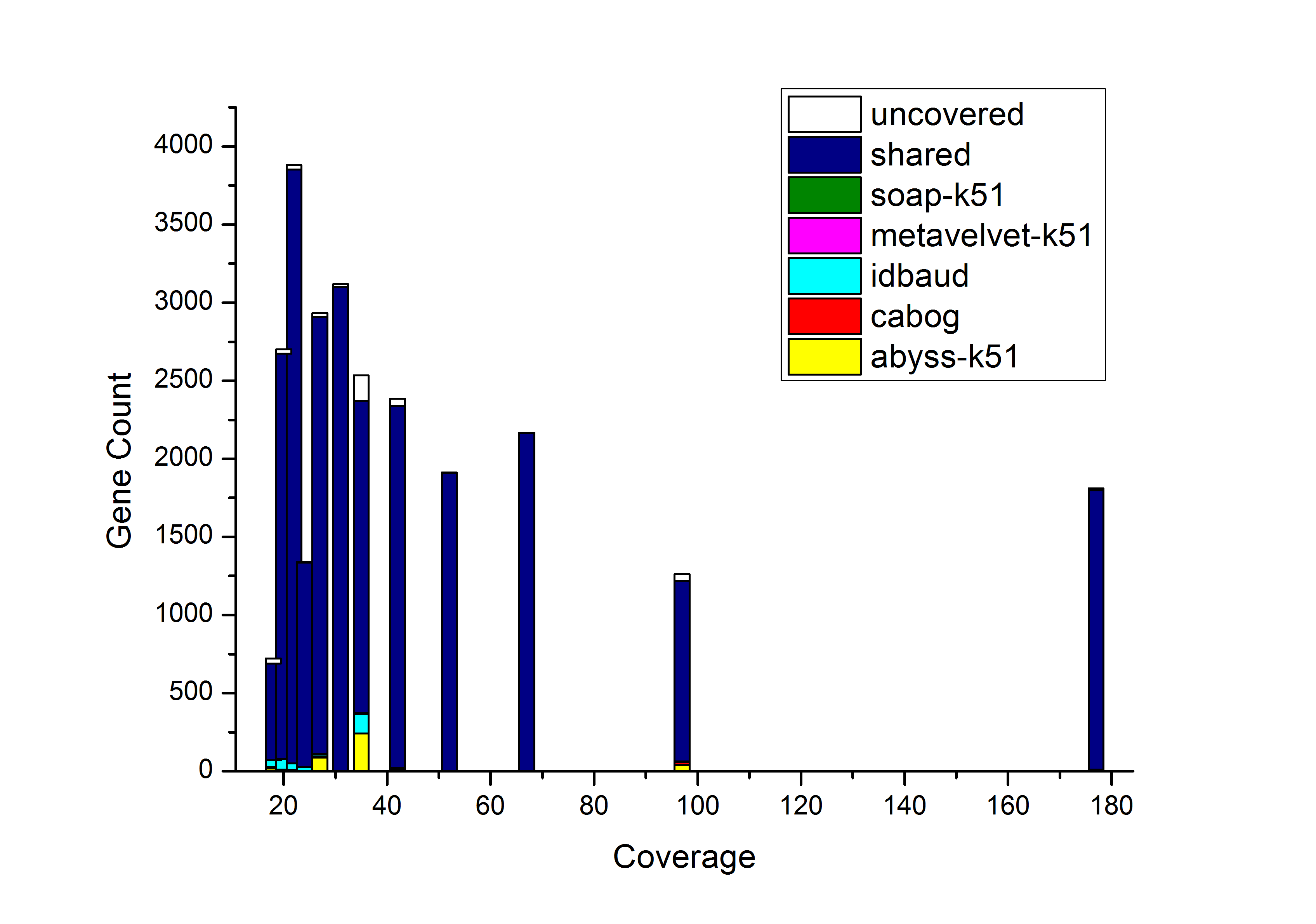


The number of genes uncovered by any assemblies (*white*), covered by more than one assemblies (*blue*) and covered exclusively by only one assembly of the five assemblers: ABySS (*k*-mer size 51) (*yellow*), CABOG (*red*), IDBA-UD (*cyan*), MetaVelvet (*k*-mer size at 51) (*magenta*), SOAPdenovo (*k*-mer size at 51) (*green*), for species with low coverage (>18×), are stacked.

**Supplemental Figure 4.** Correct *N*50 size of mixed and separate assemblies on four small simulated datasets for five assemblers.





Correct *N*50 of assemblies from five assemblers on mixed reads and separated reads from *sim1* (top left graph), *sim2* (top right graph), *sim3* (bottom left graph), and *sim4* (bottom right graph) dataset. The differences between the correct *N*50 sizes from mixed reads and from separated reads are labeled on the graphs. The *sim1* and *sim2* datasets have the same species with each belonging to different genus. The *sim3* and *sim4* datasets have the same species with each belonging to the same genus. The *sim1* and *sim3* datasets have the even coverage distribution. The *sim2* and *sim4* datasets have the uneven coverage distribution.

**Supplemental Figure 5.** Assembly error counts of mixed and separate assemblies on four small simulated datasets for five assemblers.





Error number of assemblies from five assemblers on mixed reads and separated reads from *sim1* (top left graph), *sim2* (top right graph), *sim3* (bottom left graph) and *sim4* (bottom right graph) dataset. The differences the error number between the assemblies from mixed reads and from separated reads are labeled on the graphs.

**Supplemental Figure 6.** The plot of total covered contig length versus contiguity of the assemblies generated by different assemblers on the *sim-113sp* dataset.





The assemblies plotted are from InteMAP (*black*), ABySS (*red*, with *k*-mer size at 23, 31, 41, 51 and 61), Bambus2 (*green*), CABOG (*blue*), IDBA-UD (*cyan*), SOAPdenovo (*magenta*, with *k*-mer size at 23, 31, 41 and 51, and with and without Gapcloser post-process) and MetaVelvet (*yellow*, with *k*-mer size at 23, 31, 41, 51 and 61).

**Supplemental Figure 7.** Rank-coverage curve of the simulated metagenomic dataset *sim-113sp*.


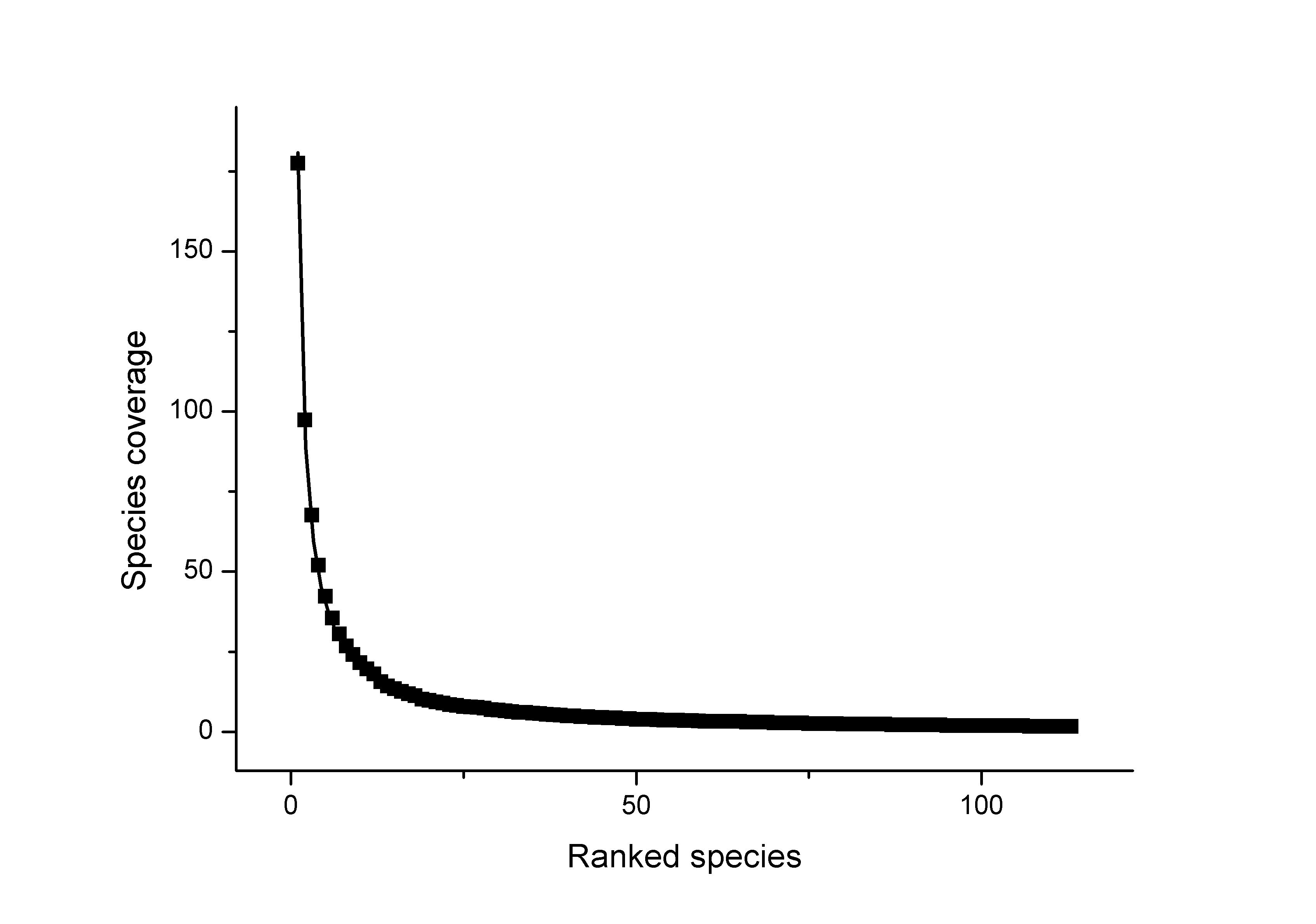


**Supplemental Figure 8.** Correct reads ratio of each species after the error correction process for dataset *sim-113sp*.


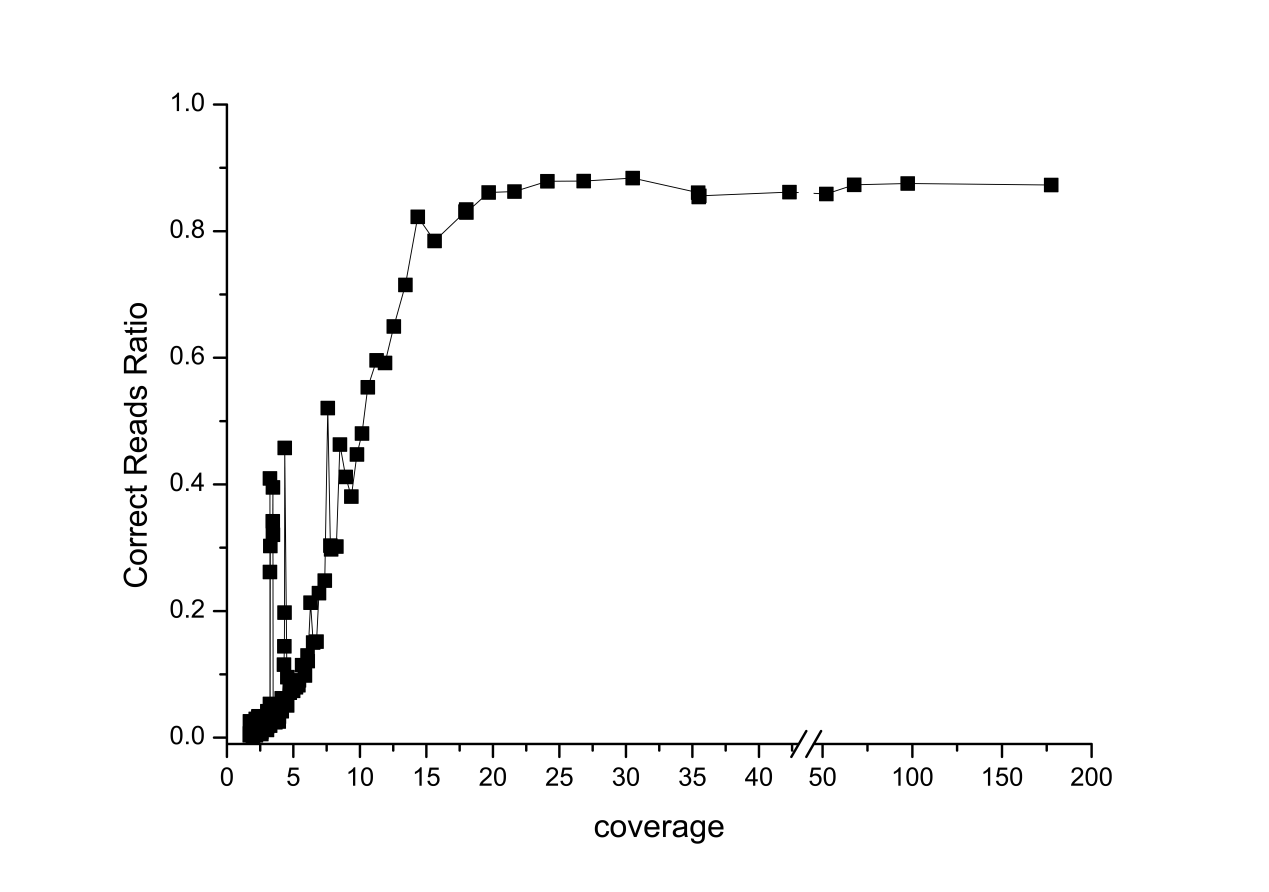

Supplement: Additional file 1: — Supplemental Materials. This file is in docx format and includes the Supplemental Methods and Supplemental Figures. (DOCX 784 kb) [file 12859_2015_686_MOESM1_ESM.docx]
